# An Intriguing Case of Pneumoperitoneum In a Patient With COVID-19: Do All Pneumoperitoneum Cases Need Surgery?

**DOI:** 10.7759/cureus.12279

**Published:** 2020-12-25

**Authors:** Munish Sharma, Pratima Ojha, Pahnwat T Taweesedt, Iqbal Ratnani, Salim Surani

**Affiliations:** 1 Internal Medicine, Corpus Christi Medical Center, Corpus Christi, USA; 2 Anesthesiology, Houston Methodist Hospital, Houston, USA; 3 Internal Medicine, University of North Texas, Dallas, USA

**Keywords:** covid 19, pneumomediastinum, pneumoperitoneum, tube thoracostomy

## Abstract

Pneumoperitoneum is pneumatosis in the potential space of the abdominal cavity. It is generally considered a surgical emergency and is mostly due to perforated hollow viscus. Rarely, pneumoperitoneum might occur even in the absence of bowel perforation. We hereby present a case of pneumoperitoneum in a patient with COVID-19 pneumonia and pneumomediastinum, which was managed non-surgically.

## Introduction

Pneumoperitoneum is characterized by the presence of abnormal gas (as air) in the potential abdominal space [1]. This usually is considered a surgical emergency and is associated with perforated abdominal viscus in more than 90% of cases [2-4]. We hereby describe a case in a patient with coronavirus disease 2019 (COVID-19) pneumonia and pneumomediastinum with pneumoperitoneum. He was treated with a wide bore chest tube for pneumomediastinum and conservatively for the pneumoperitoneum. His pneumoperitoneum resolved in five days with improvement in pneumomediastinum.

## Case presentation

**Figure 1:**
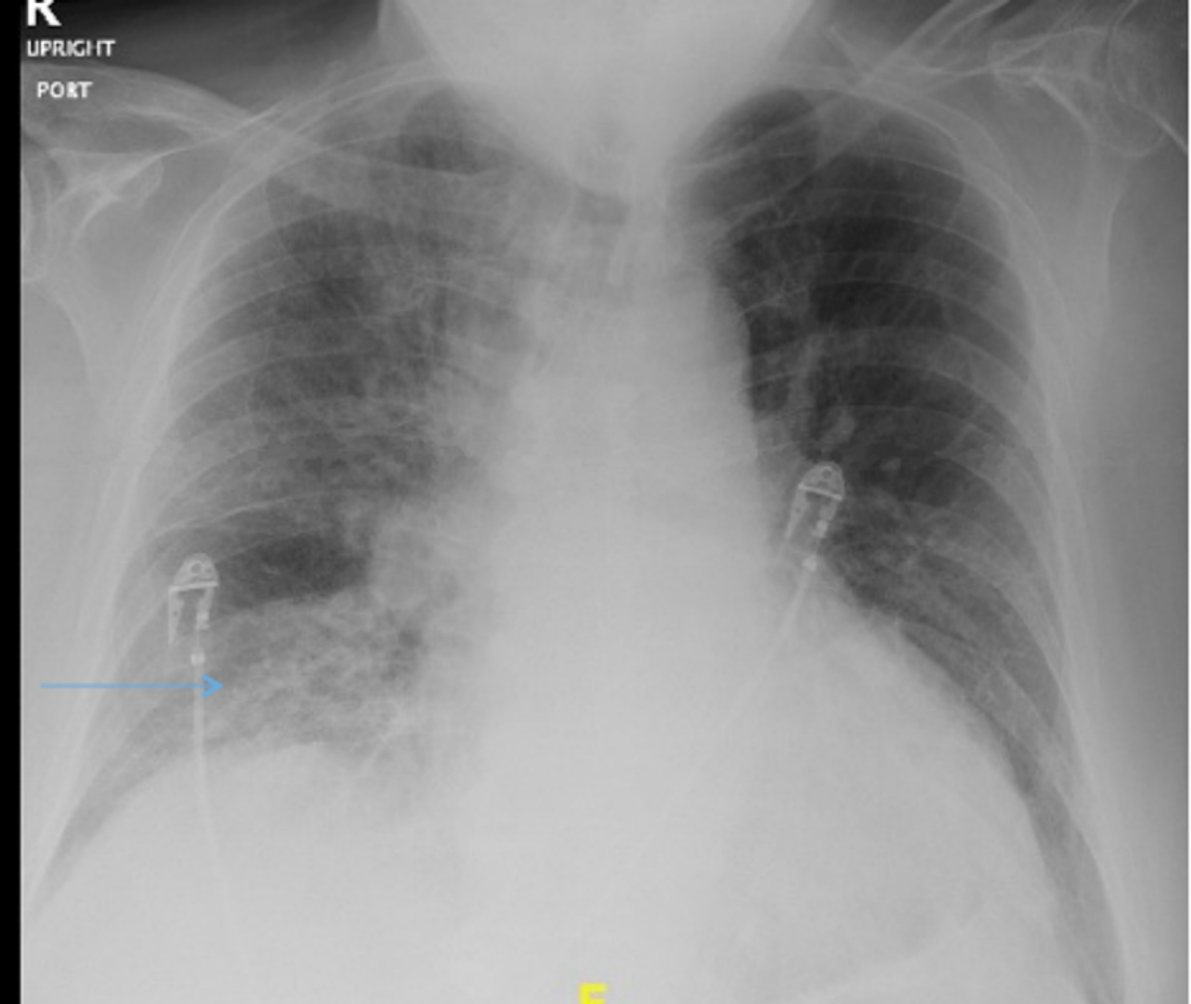
Chest X-ray showing opacities at the right lower lobe region (blue arrow)

A 47-year-old male patient with a history of hypertension, diabetes mellitus type 2, current smoker with one pack per day was admitted to our hospital with cough, congestion, shortness of breath, and fever for five days. On admission, he was found to be positive for COVID 19. His vital signs were the following: temperature 39.4° Centigrade, heart rate (HR) 124 beats/minute, blood pressure (BP) 160/110 mm Hg, oxygen saturation (SpO2) of 88% on room air, and 93% on 3L of oxygen via nasal cannula. Chest X-ray showed right pulmonary opacity mainly at the basal region with some streaky left retro cardiac opacities (Figure 1).

**Figure 2:**
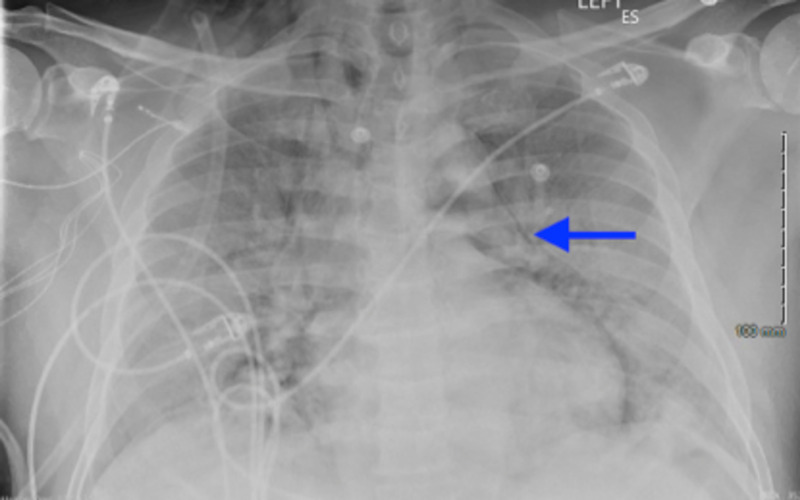
Chest X-ray showing new pneumomediastinum (blue arrow) and subcutaneous emphysema

He was able to talk in full sentences. Chest examination revealed bilateral basal crackles. Cardiac examination revealed normal heart sounds and regular rhythm, the abdomen was soft, non-tender with normal bowel sounds, and the examination of the nervous system did not reveal any abnormalities. He was started on prophylactic low dose molecular weight heparin (LMWH) subcutaneous injections, two units of convalescent plasma for COVID-19, parenteral remdesivir, and oral dexamethasone 6 mg per oral daily. Over the next seven days, the patient’s oxygen requirement increased gradually despite the treatment, and he ended up on continuous bilevel positive pressure support (BiPAP) with 100% fractional inspired oxygen (FiO2). On the ninth day of admission, his work of breathing continued to worsen even on BiPAP. His Chest x-ray showed worse diffuse bilateral airspace opacities, new pneumomediastinum, and subcutaneous emphysema (Figure 2).

**Figure 3:**
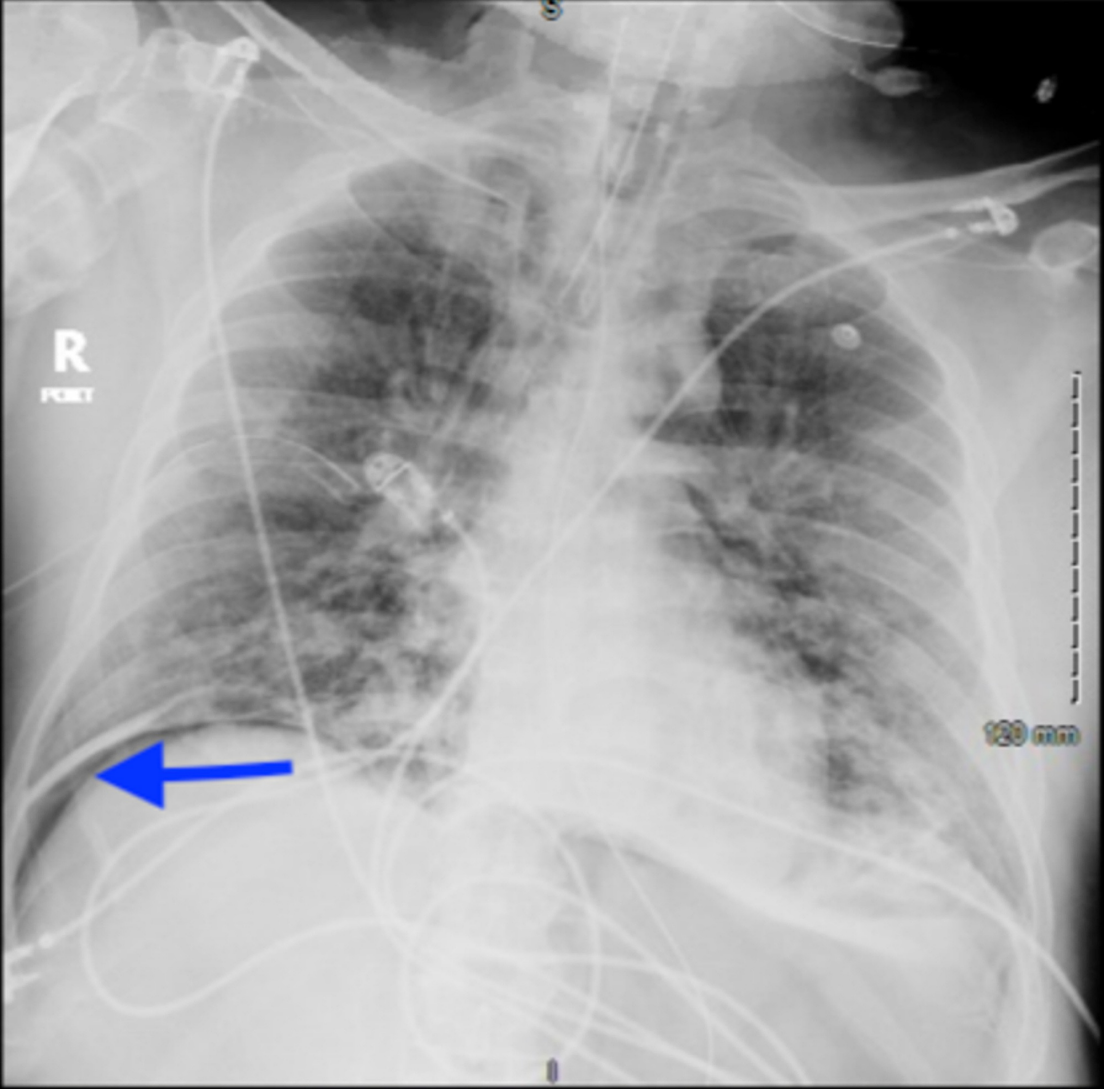
Chest X-ray showing diffuse bilateral airspace disease, pneumomediastinum, subcutaneous emphysema, and concern for free air under the diaphragm

**Figure 4:**
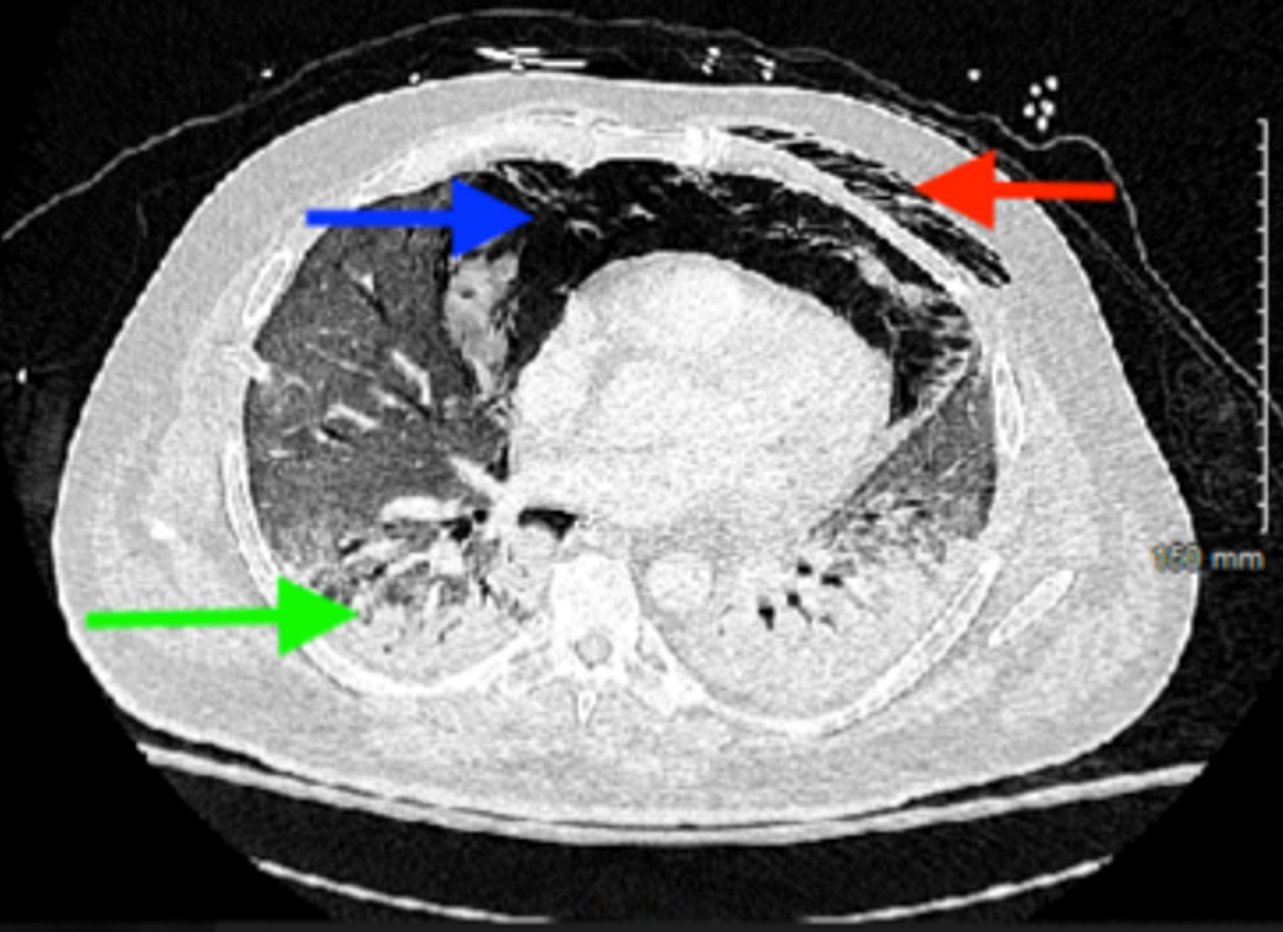
Computed tomography chest showing pneumomediastinum (blue arrow), subcutaneous emphysema (red arrow), and diffuse bilateral airspace disease (green arrow)

**Figure 5:**
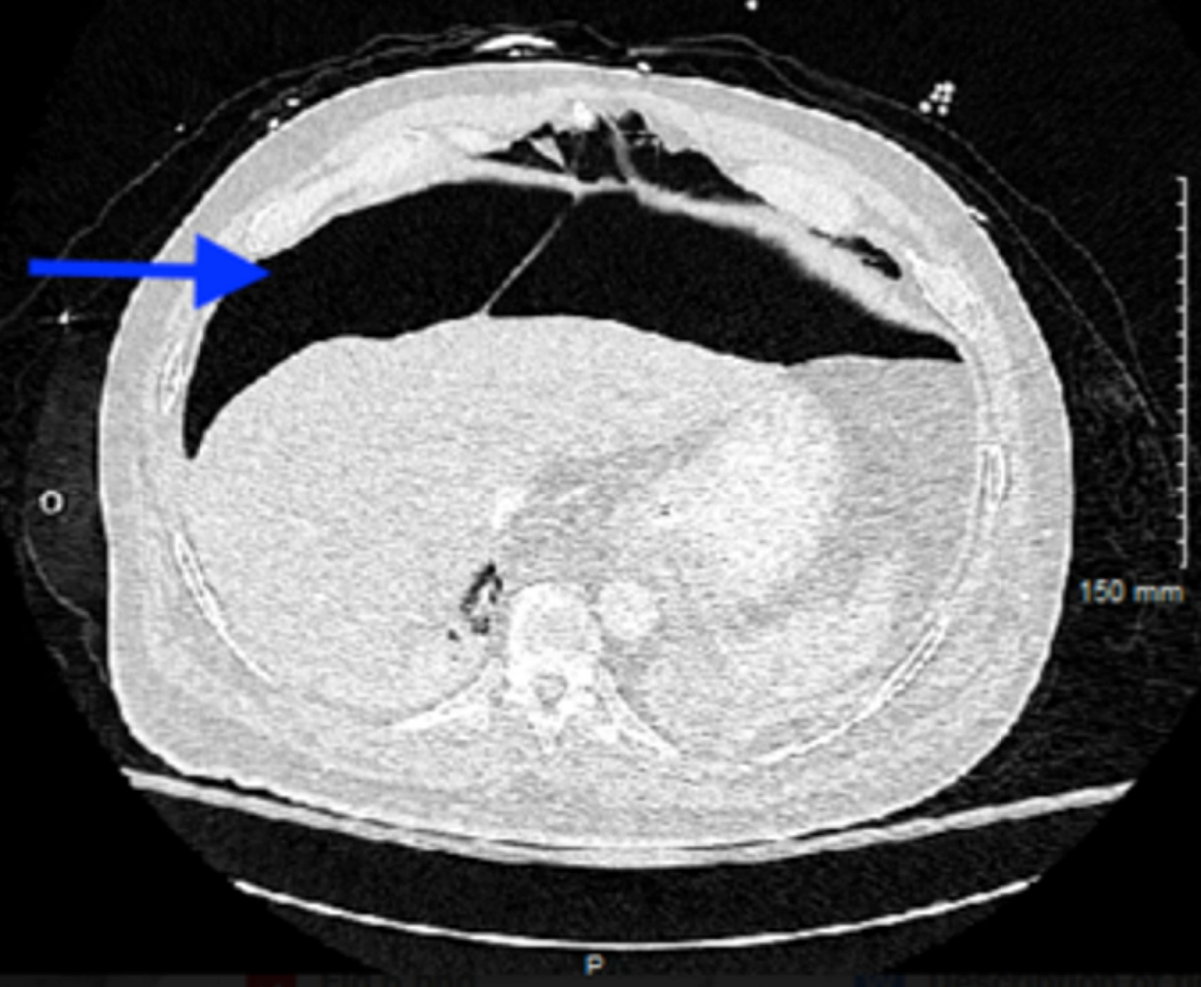
Computed tomography abdomen showing pneumoperitoneum (blue arrow)

Laboratory findings were notable for leukopenia with white blood cell count of 3.7 per microliter (range: 4.5 to 11.0 X 109 /L), worsening serum creatinine of 1.23 mg/dl (admitted with serum creatinine of 0.8 mg/dl), d-dimer 827 ng/ml (normal less than 500 ng/ml), serum ferritin 1836 ng/ml (range: 20 to 250 ng/ml), lactate dehydrogenase 363 units/liter (normal range: 140 to 280 units/ liter) and C-reactive protein of 5.3 mg/liter (normal less than 10 mg/liter). He was subsequently intubated and transferred to the intensive care unit. Given large pneumomediastinum and initiation of invasive ventilation with high positive end-expiratory pressure (PEEP) ranging from 8 to 10 centimeter of water, chest tubes were also placed preemptively. An X-ray taken the next day incidentally showed free air under the diaphragm apart from the pneumomediastinum, subcutaneous emphysema, and bilateral chest tubes (Figure 3) with similar findings in computed tomography (CT) of the chest (Figure 4). However, a CT scan of the abdomen and pelvis did not reveal any evidence of bowel perforation but did show a large pneumoperitoneum (Figure 5).

**Figure 6:**
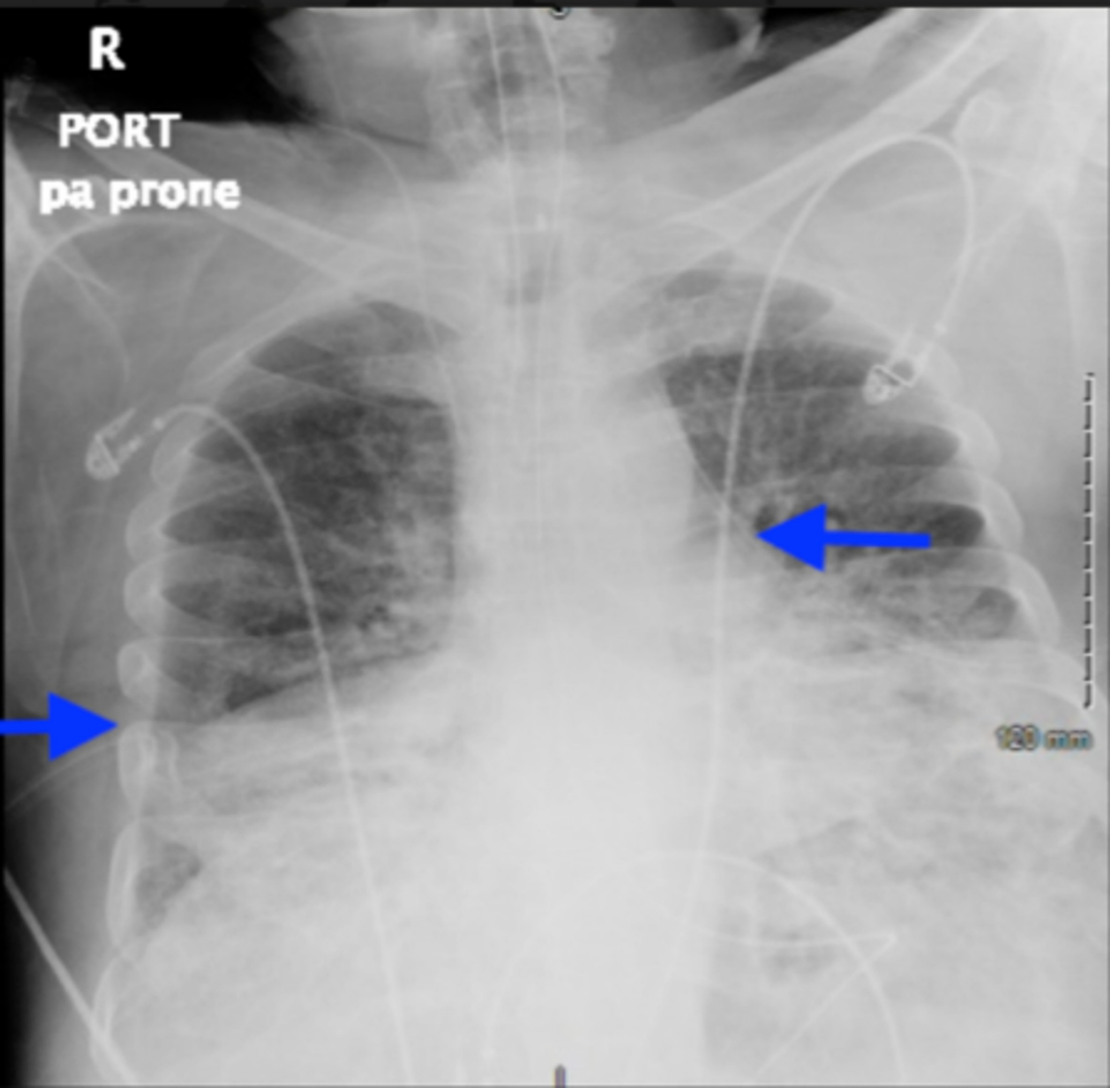
Chest X-ray taken five days after the initial diagnosis of pneumoperitoneum showing no free air under the diaphragm. Presence of bilateral chest tubes (blue arrows). Improvement in pneumomediastinum and subcutaneous emphysema.

General surgery was consulted for the pneumoperitoneum. They recommended managing the patient conservatively due to the absence of evidence for bowel perforation on computed tomography imaging of the abdomen and pelvis. The patient’s abdomen started getting softer and less distended in the next few days. Pneumoperitoneum resolved after five days of diagnosis with the improvement of pneumomediastinum (Figure 6). 

## Discussion

Pneumoperitoneum has many different causes, including a ruptured viscus, abdominal trauma, recent abdominal laparoscopy or surgery, peritoneal dialysis, paracentesis, vaginal procedures, bacterial peritonitis, pneumatosis cystoides intestinalis, and bowel malignancy [5]. 

Causes of bowel perforation can be a perforated peptic ulcer, tumor, or trauma to the abdomen. As explained earlier, more than 90% of pneumoperitoneum are associated with perforated abdominal viscus requiring emergent interventions, and up to 10% of pneumoperitoneum are due to nonsurgical etiology [5, 6]. In addition to this, high baro-pressure during mechanical ventilation is the most common thoracic cause of pneumoperitoneum [7-10]. The presence of anatomical orifices, especially in the weak areas of the diaphragm like posterolateral or parasternal, explains the passage of air from the thorax to the abdomen, also called porous diaphragm syndrome [11, 12]. These non-surgical causes may lead to unnecessary laparotomy, causing more harm over benefits but, if correctly diagnosed, can be managed successfully by conservative management alone. Our patient was also found to have a distended abdomen for two days when he was diagnosed with pneumoperitoneum. After the chest tube was inserted, the abdomen softened, and the pneumoperitoneum improved, allowing continued non-surgical management of the patient [13] posed the following question: Does pneumoperitoneum always require laparotomy? If the patient does not have any peritoneal symptoms and imaging examinations show no abdominal cavity pathology, one should consider spontaneous pneumoperitoneum, which does not require surgical intervention [14].

In our patient, despite his large pneumoperitoneum, he did not have any features suggestive of gastrointestinal tract perforation, such as nausea, vomiting, diarrhea, abdominal pain, or hemodynamic instability. He did have abdominal distension on the first two days of the radiographic findings of pneumoperitoneum, which improved gradually on the third day. This concludes that a small subset of patients with no abdominal discomforts doesn’t require immediate interventions, and incidental findings of pneumoperitoneum on X-ray abdomen or computed tomography (CT) scanning may not be of much significance. Such cases can be managed conservatively.

## Conclusions

Around 90% of patients with pneumoperitoneum require surgical intervention due to the perforation of the hollow viscus. The remaining 10% can be managed conservatively if there is no definite evidence of abdominal viscus perforation. The patient’s hemodynamic status, signs, and symptoms should be monitored carefully before coming to a diagnosis. Clinical knowledge, evidence-based practices, and clinical experience all together play a great role in the proper diagnosis and management of the patient. In the patient with COVID-19, severe adult respiratory distress syndrome related to pneumomediastinum has commonly been seen, and dissection of air from the thoracic cavity to the abdominal cavity is possible. Unnecessary surgical interventions, especially in critically ill patients with low Pao2/FiO2 ratio when not required, may cause more harm than benefit. If not associated with perforation of the alimentary tract, usually pneumoperitoneum is self-limiting.
